# Protocol for insulin dosing during expansion and maturation of human iPSC-derived cardiomyocytes

**DOI:** 10.1016/j.xpro.2026.104552

**Published:** 2026-05-18

**Authors:** Devin Verbueken, Qianliang Yuan, Vincent A.J. Warnaar, Shaima Ayubi, Keyin Zhang, Luuk J.M. Kemna, Luc Bertrand, Steven A.J. Chamuleau, Jolanda van der Velden, Jan Willem Buikema

**Affiliations:** 1 Amsterdam Cardiovascular Sciences, Department of Physiology, Amsterdam University Medical Center, VU University, 1081 HV Amsterdam, the Netherlands; 2 Department of Cardiology, Amsterdam University Medical Centers, University of Amsterdam, Amsterdam, the Netherlands; 3 UCLouvain, Intitut de Recherche Expérimentale et Clinique, Pole of Cardiovascular Research, Brussels, Belgium; 4 School of Basic Medical Sciences, Henan University, Kaifeng, China

**Keywords:** Cell culture, Stem Cells, Cell Differentiation

## Abstract

Techniques for differentiating human induced pluripotent stem cells (hiPSCs) into cardiomyocytes (hiPSC-CMs) can yield up to 98% purity, yet control over hiPSC-CM fate is lacking. Here, we present a protocol for insulin dosing during the expansion and maturation of hiPSC-derived cardiomyocytes. We describe steps for differentiation, purification, replating, cryopreservation, and thawing. We then detail a procedure for expansion using insulin and Wnt/β-catenin signaling, followed by steps to initiate maturation by either removing insulin completely or adding a low dose of insulin.

For complete details on the use and execution of this protocol, please refer to Yuan et al.^1^

## Highlights

• Instructions for the differentiation of hiPSCs into hiPSC-CMs
• Guidance on purification, replating, cryopreservation, and thawing of hiPSC-CMs
• Steps for expansion or maturation via insulin dosing and Wnt/β-catenin regulation

## Abstract (editor highlights)

Publisher’s note: Undertaking any experimental protocol requires adherence to local institutional guidelines for laboratory safety and ethics.

## Graphical abstract

**Figure:**
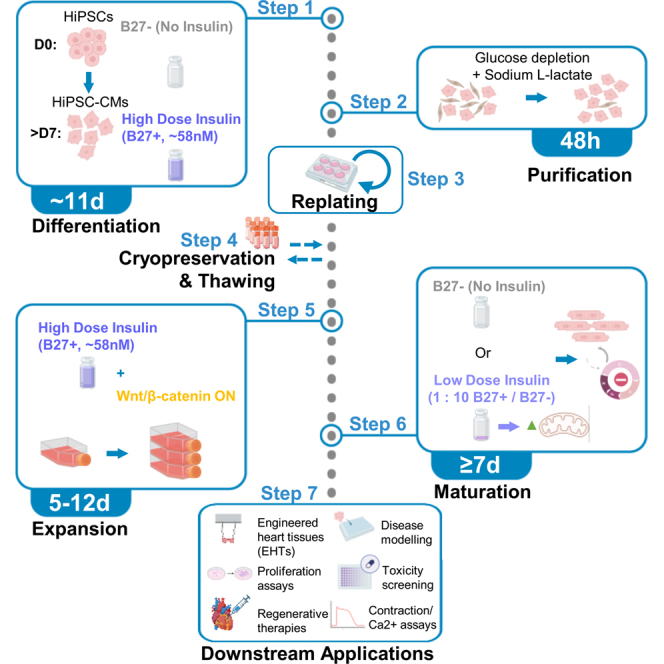

## Before you begin

Since the discovery of the Yamanaka factors to induce pluripotent stem cells from human somatic cells, various approaches have been established to differentiate human-induced pluripotent stem cells (hiPSCs) into cardiomyocytes. Lian et al.^2^ showed that through chemical stage-specific modulation of the Wnt/*β*-catenin pathway in RPMI/B27 Medium, highly pure cultures of hiPSC-CMs can be obtained (80–98% cTnT+). An overview of the different protocols and their evolution over time is summarized elsewhere.^3^

Using this RPMI/B27-directed differentiation approach, re-activation of the Wnt/*β*-catenin signaling pathway in immature hiPSC-CMs was shown to induce massive expansion, yielding a ∼250-fold increase in cell number over 3–5 passages.^4^ Recently, we have shown that the high insulin concentration in the B27+ Supplement (∼58 nM, 340 ng/mL) is required for this massive proliferative response via activation of the IGF–AKT–FOXO axis.^1^ Removal of insulin, by using B27- supplement, blunted the increase in hiPSC-CMs that are actively engaging in the cell cycle upon Wnt/*β*-catenin activation from 25- to 5-fold. Removal of both insulin and Wnt/β-catenin activation led to rapid cell cycle exit, increased RNA expression of mature sarcomeric isotypes, and enhanced sarcomeric and mitochondrial organization. Low concentrations of insulin (∼10 nM) already facilitate the translocation of GLUT4 towards the membrane in cardiac myocytes.^5^ Using a 1 in 10 mix of the B27+ and B27- supplement (1:10 B27+/B27-, ∼5.8 nM) we show that the metabolism of hiPSC-CMs is enhanced without activating the cell cycle via the IGF–AKT–FOXO axis.

In this protocol we provide an easy and cost-effective strategy for hiPSC-CM differentiation and control of resulting hiPSC-CM cell fate through modulation of the Wnt/*β*-catenin pathway and insulin dosing, and offer practical recommendations through hands-on experience.

### Innovation

In our recent work we showed that besides reactivation of the Wnt/*β*-catenin signaling pathway, the proliferative response of immature hiPSC-CMs (∼D12) also relies on a high dose of insulin (B27+, ∼58 nM) acting via the IGF–AKT–FOXO axis. The complete removal of both insulin (B27-) and Wnt/*β*-catenin activation over seven days led to immediate cell cycle exit, upregulation of adult sarcomeric mRNA isotype expression, and improved mitochondrial organization. This offers a simplified strategy to initiate maturation in hiPSC-CMs.

In this protocol, we build on these findings by showing that supplementing a low dose of insulin (1:10 B27+/B27-, ∼5.8 nM) supports the metabolism of hiPSC-CMs compared to the complete removal of insulin (B27-), without activating the cell cycle via the IGF–AKT–FOXO axis. We offer this as an alternative strategy for maturation of hiPSC-CMs whilst preventing impaired metabolism through immediate and complete loss of insulin when using B27-.

This protocol also includes practical recommendations, based on our hands-on experience using this workflow in the laboratory, to optimize culture efficiency. For example, we propose to perform hiPSC-CM purification in the presence of Wnt/*β*-catenin activator CHIR99021 to prevent quick cell cycle exit if expansion is desired afterwards. Moreover, we explain how to carefully replate newly differentiated hiPSC-CMs once before replating them for downstream applications to ensure maximum cell survival.

### Institutional permissions

All experiments using patient-derived tissues or cells must be performed under prior approval from the competent institutional ethics committee in accordance with local regulations.

### Thawing hiPSCs

**Timing: ∼12–24 h**

This section describes how to thaw hiPSCs while maintaining good cellular viability.

***Note:*** Warm all media to room temperature (RT, ∼20°C) for ≥30 min before use and coat culture plates with Matrigel (make aliquots of 1 mg/mL and dilute 1:12 with DMEM/F12) at 37°C for ≥30 min (see materials and equipment for media compositions and key resources table for all used reagents).

***Optional:*** Use mTeSR plus medium for more flexible culturing.

***Note:*** After coating, plates can be stored at 4°C for up to two weeks wrapped in parafilm to prevent drying out.

**CRITICAL:** Matrigel needs to be substituted for xeno-free and defined ECM coatings (e.g. laminins) when cells are used for clinical applications.

1. Thaw hiPSCs from liquid nitrogen.
  a. Remove a cryovial from liquid nitrogen and transfer it onto dry ice.
  b. Add 5 mL of PBS to a 15 mL tube.
    ***Note:*** For vulnerable cell lines Essential 8 or mTeSR plus medium can be used.
  c. Thaw the cryovial at 37°C until only a small shiver of ice remains, then quickly transfer the cell suspension to the tube containing PBS.
    ***Note:*** DMSO in the cryopreservation medium is toxic, so work swiftly.
  d. Centrifuge at 300 × *g* for 3 min at RT (∼20°C).
  e. Carefully aspirate the supernatant.
  f. Resuspend the cell pellet in 1 mL of hiPSC Recovering Medium.
2. Plate hiPSCs.
  a. Aspirate the Matrigel and replace with 1.5 mL hiPSC Recovering Medium per well (aim for ∼0.5–1 × 10^6^ hiPSCs per well).
    ***Note:*** Make sure that the plate is not too confluent, since this may result in loss of pluripotency.
  b. Gently rock the plate back and forth to distribute the cells evenly.
  c. Replace the medium after 12–24 h with 2 mL E8 and thereafter replace medium every 24 h.
    ***Optional:*** Replace with 2 mL mTeSR plus medium and replace every 48 or 96 h (double medium).

### Passaging hiPSCs

**Timing: ∼2 weeks**

Here we describe how hiPSCs can be passaged to maintain a healthy proliferative culture before initiating differentiation.

***Note:*** Passage hiPSCs for at least three times prior to initiating directed differentiation towards hiPSC-CMs.

***Note:*** Ideally, hiPSCs are cultured for 4–5 consecutive days to achieve 85%–90% confluency, before initiating differentiation. This way, you ensure that hiPSCs are healthy and actively proliferating, while not overgrown, which could lead to reduced proliferation and spontaneous differentiation.

**Figure 1:**
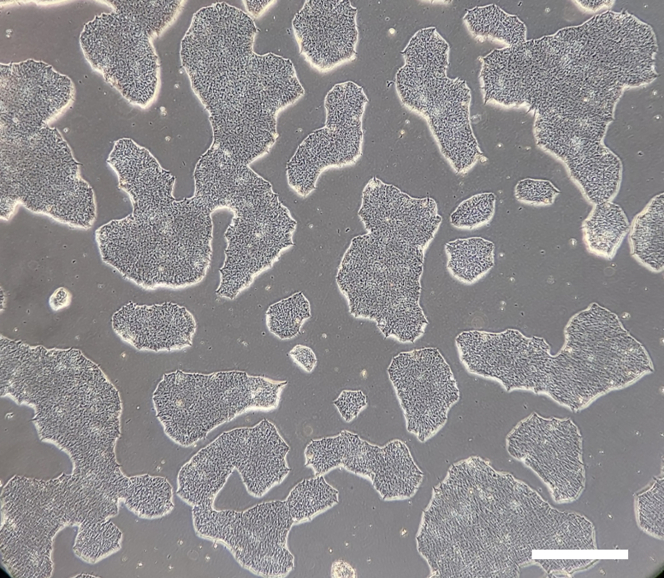
Representative image of SCVI-114 hiPSCs with ∼70% confluency ready for passaging Scale bar, 500 μm.

3. When the culture reaches ∼70% confluency, proceed with passaging (Figure 1).
  ***Note:*** If hiPSCs reach too high confluency, loss of pluripotency may occur.
  ***Note:*** Handle hiPSCs quickly, minimize the amount of time that they are outside of the incubator.
  a. Aspirate the culture medium and add 750 μL EDTA per well of a 6-well plate; incubate for 3 min at RT (∼20°C).
    **Note:** To assess detachment, gently pipette EDTA over the cell layer. If cells remain adherent, incubate for 1 additional minute and test again.
  b. Add 1.5 mL of hiPSC Recovering Medium to a new Matrigel-coated well.
    **CRITICAL:** Matrigel needs to be substituted for xeno-free and defined ECM coatings (e.g. laminins) when cells are used for clinical applications.
  c. Aspirate the EDTA solution and gently resuspend the cells in 1 mL of hiPSC Recovering Medium.
    ***Note:*** Dissociate cells in 5 to 6 times, to create small aggregates of cells without inducing too much cell damage.
  d. Transfer the desired volume of cell suspension into the prepared well.
    ***Note:*** Aim for ∼70% confluency within 3–4 days to maintain hiPSCs healthy and proliferating.
  e. Replace E8 Medium every 24 h.
    ***Optional:*** When using mTeSR plus replace every 48 or 96 h (double medium).
  f. Once cultures reach ∼70% confluency, repeat passaging as described above (step 3).

## Key resources table

**Table:** 

REAGENT or RESOURCE	SOURCE	IDENTIFIER
**Chemicals, peptides, and recombinant proteins**		

RPMI-1640 Medium	Thermo Fisher Scientific	Cat# 11875093
B-27 Supplement (50×), serum free	Thermo Fisher Scientific	Cat# 17504044
B-27 Supplement (50×), minus insulin	Thermo Fisher Scientific	Cat# A1895601
Essential 8 Medium	Thermo Fisher Scientific	Cat# A1517001
mTeSR Plus	Stemcell Technologies	Cat# 100-0276
Trypsin-EDTA (0.5%), no phenol red	Gibco	Cat# 15400054
TrypLE™ Select Enzyme (10×), no phenol red	Gibco	Cat# A1217701
Sodium L-lactate	Sigma Aldrich	Cat# 71718
Matrigel Growth Factor Reduced (GFR)^a^	Corning	Cat# 354230
DMEM/F-12	Gibco	Cat# 11320033
Penicillin-Streptomycin	Gibco	Cat# 15140122
PSC Cryopreservation Kit	Gibco	Cat# A2644601
CHIR 99021	Tocris	Cat# 4423
Wnt-C59	R&D Systems	Cat# 5148
RPMI 1640 Medium, no glucose	Gibco	Cat# 11879020
KnockOut™ Serum Replacement	Gibco	Cat# 10828028
Y-27632	Stemcell Technologies	Cat# 129830-38-2
RevitaCell™ Supplement (100×)	Gibco	Cat# A2644501

**Experimental models: Cell lines**		

SCVI-111	Stanford University	
SCVI-114	Stanford University	
SCVI-273	Stanford University	

**Deposited data**		

RNA-seq data associated published manuscript	Gene Expression Omnibus - NCBI	GSE278598

**Software and algorithms**		

Biorender	Biorender.com	

**Other**		

Seahorse XF Analyzer	Agilent	
Eclipse Ti2 Microscope	Nikon	

a When cells are used for clinical applications, Matrigel needs to be substituted for xeno-free and defined ECM coatings such as laminins.

## Materials and equipment

### HiPSC culture media

**Table:** HiPSC Recovering Medium

Reagent	Final concentration	Amount
Essential 8 basal medium	1×	As required
Essential 8 supplement	1×	1 : 50
Y-27632 (10 mM)	5 μM	1 : 2000

Store at 4°C for up to 2 weeks after preparation.

***Note:*** The dilution of Y-27632 can be adjusted to optimize cell attachment.

***Optional:*** To reduce frequency of medium changes, mTeSR Plus can be used. Replace medium every 48 h or 96 h (double medium).

### HiPSC-CM culture media

**Table:** HiPSC-CM Purification Medium A

Reagent	Final concentration	Amount
RPMI 1640 Medium, no glucose	1×	As required
B-27 Supplement (50×), serum free	1×	1 : 50
Sodium L-Lactate	5 mM	N/A
CHIR 99021 (10 mM)	2–4 μM	1:2500 – 1:5000

Store at 4°C for up to 2 weeks after preparation.

**Table:** HiPSC-CM Purification Medium B

Reagent	Final concentration	Amount
RPMI 1640 Medium, no glucose	1×	As required
B-27 Supplement (50×), minus insulin	0.9×	9:500
B-27 Supplement (50×), serum free	0.1×	1:500
Sodium L-Lactate	5 mM	N/A

Store at 4°C for up to 2 weeks after preparation.

**Table:** HiPSC-CM Replating Medium

Reagent	Final concentration	Amount
RPMI-1640	1×	As required
B-27 Supplement (50×), serum free	1×	1:50
Y-27632 (10 mM)	5 μM	1:2000

Store at 4°C for up to 2 weeks after preparation.

***Optional:*** 1 : 100 Revitacell can be used instead of Y-27632 for fragile cell lines.

#### Step 5: Expansion

**Table:** Expansion medium

Reagent	Final concentration	Amount
RPMI-1640 Medium	1×	49 mL
B-27 Supplement (50×), serum free	1×	1 mL
CHIR-99021 (10 mM)	2–4 μM	10 – 20 μL

Store at 4°C for up to 2 weeks after preparation.

***Optional:*** 1% Penicillin/Streptomycin.

***Note:*** The optimal CHIR-99021 concentration varies per cell line. Test a range of concentrations to identify the most effective dose for maximal expansion.

#### Step 6: Maturation

**Table:** RPMI(B27-) No Insulin

Reagent	Final concentration	Amount
RPMI-1640 Medium	1×	49 mL
B-27 Supplement (50×), minus insulin	1×	1 mL

Store at 4°C for up to 2 weeks after preparation.

***Optional:*** 1% Penicillin/Streptomycin.

**Table:** RPMI(1:10 B27+ / B27-) Low Dose Insulin

Reagent	Final concentration	Amount
RPMI-1640 Medium	1×	50 mL
B-27 Supplement (50×), minus insulin	0.9×	900 μL
B-27 Supplement (50×), serum free	0.1×	100 μL

Store at 4°C for up to 2 weeks after preparation.

***Optional:*** 1% Penicillin/Streptomycin.

## Step-by-step method details

***Note:*** All reagents can be found in the key resources table and media compositions are provided in materials and equipment. Bring all used media to room temperature (RT, 20°C) for ≥30 min prior to use.

### Differentiation into hiPSC-CMs

**Timing: 9 days**

After this step, a highly pure and contractile hiPSC-CM culture can be achieved.

**Figure 2:**
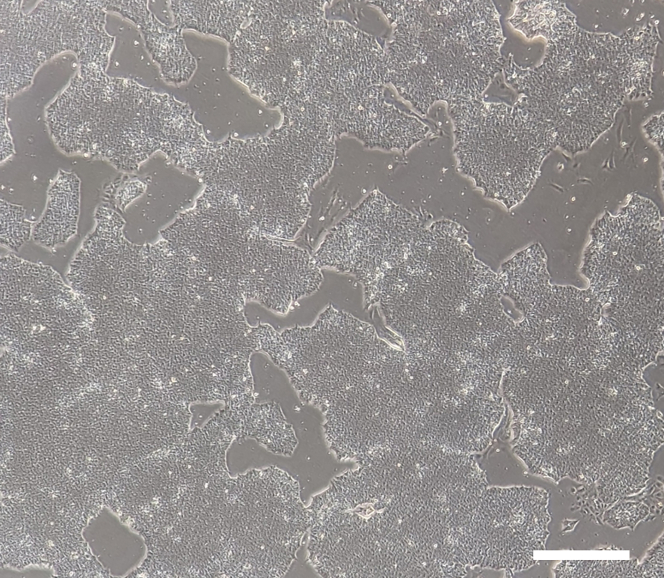
SCVI-114 hiPSCs with ∼85%–90% confluency, optimal for mesoderm induction Scale bar, 500 μm.

***Note:*** When cells reach high confluency in < 3 days, avoid differentiation. This typically indicates that cells were plated too densely. Evenly distributed colonies are required to ensure optimal mesoderm induction.

***Note:*** To increase consistency and robustness of differentiation, aim to change culture medium the same time every day.

1. Prepare hiPSCs for differentiation.
  a. Culture hiPSCs in a 6 well plate until they reach 85–90% confluency (Figure 2). Ideally hiPSCs are cultured for 4–5 consecutive days before reaching this confluency.

2. Continue with differentiation toward hiPSC-CMs.
  Day 0.
  a. Replace the medium with 3 mL of RPMI/B27(INS-) supplemented with 4 – 10 μM CHIR99021.
    **CRITICAL:** Determine the optimal CHIR99021 concentration for each cell line and passage number by testing a gradient between 4 and 10 μM.
    ***Note:*** If testing a broader range of CHIR99021 is warranted, a 12 or 24-well plate can be used with medium volumes of 2 and 1 mL respectively.
    ***Note:*** Handle plates carefully during this step; cells are highly sensitive.
    Day 2.
  b. After 48 h, replace the medium with RMPI/B27(INS-).
    ***Note:*** For best results, perform this medium change as close as possible to exactly 48 h after adding CHIR99021.
    Day 3.
  c. Replace medium after 24 h with 3 mL RPMI/B27(INS-) containing 2 μM Wnt-C59.
    Day 5.
  d. Replace medium with 3 mL RPMI/B27(INS-) after 48 h.
    Day 7.
  e. After 48 h, replace medium with 3 mL RPMI/B27(INS+).
    Day 9.
  f. HiPSCs should be differentiated towards immature hiPSC-CMs. In some cases they already display contraction.
    ***Note:*** If morphology is deviant from the example (Methods video S1), consult problem 1.

### Purification (<80% purity)

**Timing: 48–96 h**

Optional step to enhance the purity of the hiPSC-CM culture via metabolic selection.

3. Estimate the purity of your differentiation by assessing the percentage of contractile versus non-contractile area.
  ***Note:*** If purity is low, first consult problem 2 and then decide if you want to perform purification.
  a. Purification before *Step 5: Expansion*.
    i. Aspirate the culture medium from wells.
    ii. Add 2 mL of *Purification Medium A* to the wells.
    iii. Incubate for 48 h at 37°C and 5% CO2.
    iv. Reassess the purity, repeat sub-step 3a if purity is still <80%.
  b. Purification before Maturation (*Step 6: Maturation*).
    i. Aspirate the culture medium from wells.
    ii. Add 2 mL of *Purification Medium B* to the wells (see materials and equipment).
    iii. Incubate for 48 h at 37°C and 5% CO2.
    iv. Reassess the purity, repeat sub-step 3 b if purity is still <80%.

### Replating

**Timing: ∼1–2 h + 24 h attachment.**

We here describe how to replate hiPSC-CMs into a monolayer prior to continuing with further steps.

**CRITICAL:** Continuance into the other steps requires cells to be replated. Even if cells will be directly used for downstream applications, replating once to achieve a homogenous monolayer is generally advised.

4. Prepare culture plates for replating.
  a. Coat the desired plate with Matrigel for ≥30 min at 37°C in the incubator (make aliquots of 1 mg/mL and dilute 1:12 with DMEM/F12).

**CRITICAL:** Matrigel needs to be substituted for xeno-free and defined ECM coatings (e.g. laminins) when cells are used for clinical applications.

***Note:*** Make sure to cover the bottom of the entire plate.

***Note:*** Replate into a 6-well plate for *Maturation* (*Step 6: Maturation*, ∼100,000 hiPSC-CMs per cm^2^), and a T75 for *Expansion* (*Step 5: Expansion*, ∼10,000 hiPSC-CMs per cm^2^).

***Note:*** After coating, plates can be stored at 4°C for up to two weeks wrapped in parafilm to prevent drying out.

5. Detach hiPSC-CMs and prepare culture plates.
  a. Aspirate medium from the culture plate and replace with 0.75 mL TrypLE (10×) per well of a 6 well plate to detach the cells and incubate for 10 min at 37°C and 5% CO2.
    ***Note:*** After incubation, check whether cells detached through shaking the plate. If not, place back in the incubator and check again in 5 min. Repeat until all cells are detached up to a maximum of 30 min.
  b. During incubation, aspirate Matrigel from the pre-coated plate and replace it with hiPSC-CM Replating Medium.
    ***Note:*** Use 2 mL for a well of a 6 well plate, and 10 mL for a T75.
6. Plate hiPSC-CMs.
  a. Add 2× the volume of RPMI/B27(INS+) directly to the TrypLE (do not mix) and gently transfer the suspension to a 15 mL tube.
  b. Centrifuge at RT (20°C) for 3 min at 300 × *g* until a solid pellet is formed.
    ***Note:*** Check if a solid pellet has formed. If not, repeat centrifugation.
  c. Aspirate the supernatant carefully until only a pellet remains.
  d. Using a P1000 pipet to dissolve the pellet in 1 mL of hiPSC-CMs Replating Medium, mix thoroughly and transfer to the plate of choice in the desired ratio.
    ***Note:*** Tryphan Blue staining can be used to count the number of cells and determine replating ratio if desired.
  e. Carefully place the plate(s) in the incubator and, when in place, move back and forth and side to side to distribute cells equally over the plate.
  f. Incubate for ≥24 h to allow cells to fully attach.
    ***Note:*** See Methods video S2 for reference of a successfully replated monolayer of hiPSC-CMs.

### Cryopreservation and thawing

**Timing: Between P1 and P3, ∼24 h**

This step describes how to cryopreserve and thaw your hiPSC-CMs whilst maintaining cell viability.

**CRITICAL:** To maintain post-thaw cell viability and sufficient plate attachment, hiPSC-CMs should be passaged minimally one to maximally three times.

***Note:*** It is also possible to cryopreserve after one round of *Step 5: Expansion*, but not after *Step 6: Maturation*.

7. Cryopreservation.
  a. Place a Mr. Frosty and the required number of cryovials at 4°C ≥30 min before cryopreservation.
  b. Aspirate medium from the culture plate and replace with 0.75 mL TrypLE (10×) per well of a 6 well to detach cells.
  c. Incubate for 10 min at 37°C and 5% CO2.
    ***Note:*** Check if cells detach when shaking, if not, place them back in the incubator for 5 min at 37°C 5% CO2. Repeat as many times as necessary or 30 min is reached.
  d. Add 2× the volume of RPMI/B27(INS+) directly to the TrypLE (do not mix) and gently transfer the suspension to a 15 mL tube.
  e. Centrifuge at RT for 3 min at 300 × *g* until a solid pellet is formed.
    ***Note:*** Check if a solid pellet has formed. If not, repeat the centrifuge step.
  f. Aspirate the supernatant carefully until only a pellet remains.
  g. Carefully add 0.5–1 mL of cold PSC Cryopreservation Medium and mix until homogenous, and transfer to cryovials.
    **CRITICAL:** Keep PSC cryopreservation Medium and cryovials always on ice.
    ***Note:*** We advise to use 0.5 mL of PSC cryopreservation Medium per 5 × 10^6^ hiPSC-CMs.
  h. Transfer the cryovials to the Mr. Frosty and store at −80°C ∼24 h before transferring to liquid nitrogen.
    **Pause point:** HiPSC-CMs can be stored in liquid nitrogen for long term and thawed (sub-step 8) when cells are required for your required downstream applications.
8. Thawing.
  a. Coat the desired plate with Matrigel for ≥30 min at 37 °C.
  b. Aspirate the Matrigel from a pre-coated plate and replace with hiPSC-CM Replating Medium.
    ***Note:*** Use 2 mL Replating Medium for a well of a 6-well plate, and 10 mL Replating Medium for a T75.
  c. Remove a cryovial from liquid nitrogen and transfer it onto dry ice.
  d. Add 5 mL of RPMI/B27(INS+) to a 15 mL tube.
  e. Thaw the cryovial at 37°C until only a small shiver of ice remains, then quickly transfer the cell suspension to the tube containing RPMI/B27(INS+).
    **CRITICAL:** Keeping the hiPSC-CMs for too long at RT is toxic due to DMSO in the PSC Cryopreservation Medium.
  f. Centrifuge at 300 × *g* for 3 min.
  g. Carefully aspirate the supernatant until a pellet is left.
  h. Resuspend the cell pellet in 1 mL of hiPSC-CM Replating Medium.
  i. Plate the required volume into a Matrigel-coated plate.
  j. Gently rock the plate back and forth and side to side to distribute the cells evenly.
  k. Replace the medium after ≥12 h with RPMI/B27(INS+).

### Expansion

**Timing: 5–12 days per round (1–3 rounds)**

The goal of this step is to achieve a significant increase in hiPSC-CM cell count. 1–2 rounds of expansion may result in a ∼20–50 fold increase in cell count.

**CRITICAL:** Not possible after *Step 6: Maturation*.

***Note:*** To make sure that there are no cell-cell contacts, we recommend replating one well of a 6 well plate into a T75 flask, or ∼2 × 10^6^ hiPSC-CMs directly after thawing.

***Note:*** Expansion efficiency decreases with each round; the exponential growth rate declines progressively.

9. Expansion of hiPSC-CMs.
  a. Replace the medium with hiPSC-CMs Expansion Medium (13 mL per T75).
  b. Refresh the Expansion Medium every 2–3 days until culture reaches full confluency.
  c. To continue expansion, replate sparsely into new plates (from one T75 into three new T75 flasks) (see *Step 3: Replating*).

***Note:*** Consult problem 3 if expansion of hiPSC-CMs during this step is limited.

### Maturation

**Timing: ≥ 7 days**

During this step, maturation of hiPSC-CM is initiated, either with or without stimulation of baseline metabolism. HiPSC-CMs typically achieve improved genetic, metabolic, and functional maturation within one week.

**CRITICAL:** After maturation, *Step 5: Expansion* is not possible anymore.

10. Determine if you want to perform Maturation with (sub-step 12) or without (sub-step 11) stimulation of metabolic activity.
11. Initiate maturation of hiPSC-CMs through complete removal of insulin.
  a. Replace the medium with 3 mL per well of a 6 well plate of RPMI/B27(INS-) No Insulin.
  b. Refresh medium every 48 h for ∼7 days to allow hiPSC-CMs to mature.
12. Initiate maturation of hiPSC-CMs while stimulating metabolic activity with a low dose of Insulin.
  a. Replace the medium with 3 mL per well of a 6 well plate with RPMI(1:10 B27+/B27-) Low Dose Insulin.

**Figure 3:**
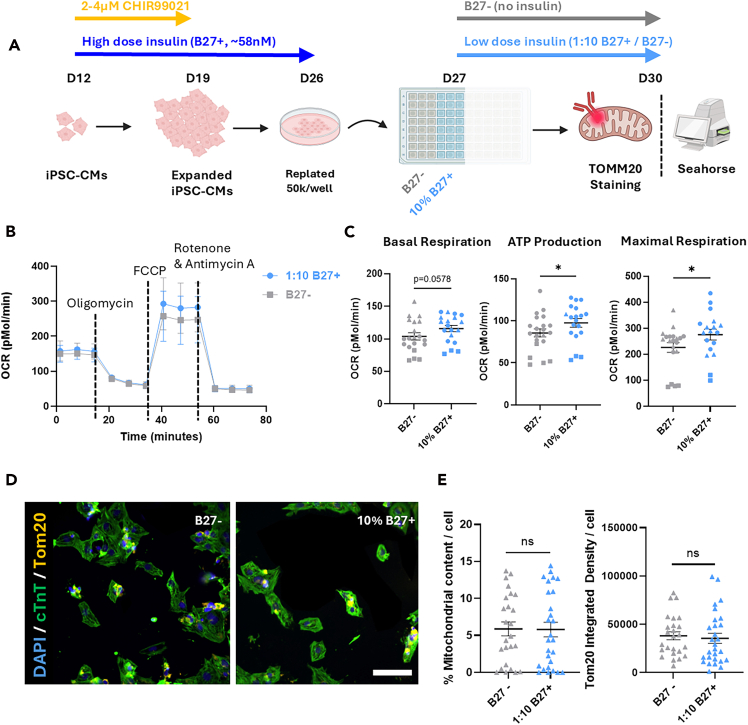
The effect of Low dose insulin (1:10 B27+/B27-, ∼5.8 nM) on oxygen consumption rate and mitochondrial content (A) Schematic overview of the experimental setup. Directly after differentiation, hiPSC-CMs were expanded for seven days, whereafter they were replated in a seahorse plate and treated with no (B27-) or low dose (1 : 10 B27+/B27-) insulin for four days. (B) Oxygen consumption rate (OCR) over time after treatment with subsequently oligomycin, FCCP, and roteone & antimycin A. Every dot represents the average of n=8 biological replicates of three different cell lines (SCVI-273, SCVI-114 & SCVI-111). (C) Derived parameters of seahorse analysis; Basal Respiration, ATP Production, and Maximal Respiration (SCVI-273 = ▲, 114 = •, and 111 = ▪). Data represents mean and SEM. (D) Representative immunofluorescence images of hiPSC-CMs. Cells were stained for nuclei (DAPI, blue), cardiac troponin T (cTnT, green), mitochondria (TOM20, yellow). Scale bar = 100μm E. Percentage mitochondrial content and integrated density of TOM20 per cell. Every triangle represents the analysis of one image from n=8 biological replicates from the SCVI-273 cell line. Data represents mean and SEM. Normality was assessed by visual inspection of Q–Q plots and by Shapiro–Wilk tests, after which independent t-tests were performed. ns = not significant, ^∗^*p* ≤ 0.05.

**Figure 4:**
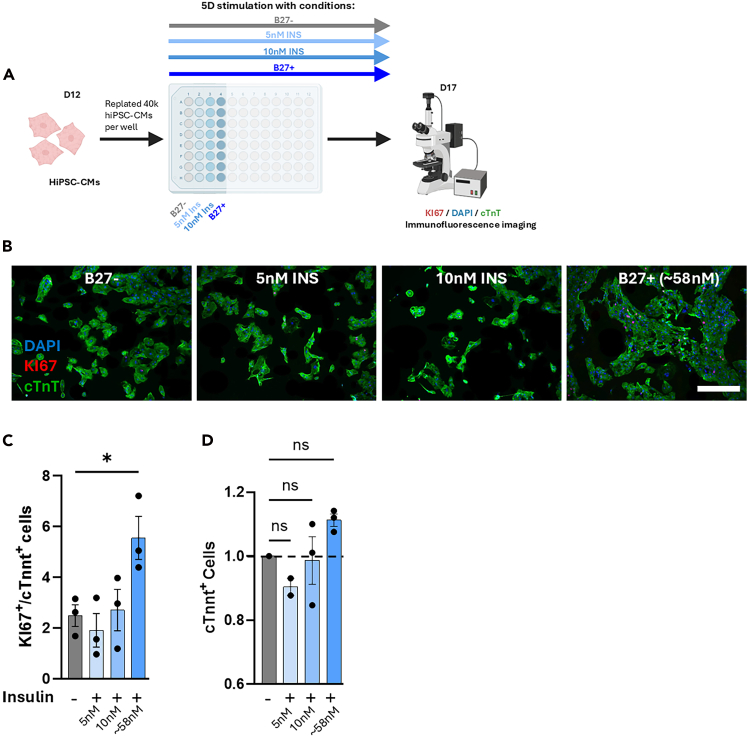
Cell cycle activity in hiPSC-CMs cultured with no, low dose (5–10 nM), and high dose (∼58 nM) insulin (A) Schematic overview of the experimental setup. D12 hiPSC-CMs were plated into wells of a 96 well plate, whereafter they were cultured with no (B27-), low dose (5 or 10 nM), or high dose (B27+) insulin for five days whereafter immunofluorescence imaging was performed. (B) Representative immunofluorescence images of hiPSCs stained for nuclei (DAPI, blue), cardiac troponin T (cTnT, green), and proliferation marker KI67 (red). Scale bar = 100μm. (C) Fraction of Ki67+-hiPSC-CMs and total number of cTnT+ hiPSC-CMs. Data represent mean and SEM. Every dot represents the average of ∼6 images from one well. HiPSC-cms are derived from the SCVI-114 line. Data was tested for normality using Pearson D’Agostino’s K-squared test, analysis of QQ-plots and significance was determined with One-Way ANOVA, followed by Tukey’s multiple comparisons test. ns = not significant, ∗*p* ≤ 0.05.

***Note:*** For detailed data underlying this recommendation refer to Figures 3 and 4.

***Note:*** If hiPSC-CMs are not viable during maturation, consult problem 4.

***Note:*** Matured cells can subsequently be used for *Step 7: Downstream applications of hiPSC-CMs*. For functional maturity on the protein level we advise to continue with metabolic maturation strategies (e.g. Feyen et al^6^), or more advanced 3D/Co-culture strategies.

### Downstream applications of hiPSC-CMs

Resulting hiPSC-CMs with the required (im)maturity can be used for downstream applications.

***Note:*** Immature hiPSC-CMs still maintain proliferative capacity and can therefore be used for proliferation assays, simple drug toxicity screening models, formation of engineered heart tissues (EHT), or potentially regenerative therapies. In this context, their fetal-like phenotype can be advantageous, as it enables rapid cell expansion and tissue assembly. Moreover, immature hiPSC-CMs are well suited for studies focusing on early developmental processes or cell cycle regulation, where full adult maturation is not required.

***Note:*** More mature hiPSC-CMs can be used to more accurately reflect the adult myocardium. The level of maturation required depends strongly on the specific research question. For example, certain disease-causing mutations affect sarcomeric proteins or isoforms that are predominantly expressed in adult cardiomyocytes, requiring sufficient maturation to ensure their proper expression. Similarly, studies assessing drug effects on cardiomyocyte mitochondrial function require an adult-like mitochondrial organization and substrate utilization, as immature hiPSC-CMs have poorly developed mitochondria and rely on glucose as a substrate.

## Expected outcomes

After differentiation of hiPSCs into hiPSC-CMs, a highly pure contractile multilayered hiPSC-CM culture should have formed (Methods video S1). If hiPSC-CMs are less pure and display only a low contractile area (<80%), *Step 2: Purification (<80% purity)* should be considered. For all further downstream applications *Step 3: Replating* is required; Either sparsely for *Step 5: Expansion* (∼10,000 hiPSC-CMs per cm^2^*)* or for *Step 6: Maturation* into a fully confluent monolayer of contractile hiPSC-CMS (∼100,000 hiPSC-CMs per cm^2^ (Methods video S2)).

*Step 5: Expansion*: In our previous work we showed that upon the administration of both high dose insulin (∼58 nM) and Wnt/β-catenin activator CHIR99021 (2 – 4 μM) over seven days, the Ki67 expression increased with ∼25-fold, and the number of hiPSC-CMs with ∼4-fold.^1^ This is in line with previous work from Buikema et al.^4^ that showed that activating Wnt/β-catenin signaling, together with sparse replating, could reach subsequently 8-, 8-, and 4-fold increases in cell numbers over three passages, resulting in a robust proliferative response of hiPSC-CMs for a maximum of 1–4 weeks.

*Step 6: Maturation*: We showed that complete removal of insulin and Wnt/β-catenin activators from the culture media for only 5 – 7 days resulted in an immediate loss of cell cycle activity (∼1% engaging in the cell cycle).^1^ Moreover, mRNA expression of adult sarcomeric genes was increased (*MYH7/6* ratio, *TNNI3* level), as well as mitochondrial organization, depicting a more mature phenotype.

Adding to this data, we here show that the use of low dose insulin (1 : 10 B27+/B27 -, ∼5.8 nM insulin) supports the metabolism of maturing hiPSC-CMs without activating the IGF–AKT–FOXO signaling pathway. Supplementing hiPSC-CMs after one round of Expansion (*Step 5*) with low dose insulin exhibited enhanced baseline respiration, ATP production, and maximal respiration compared to hiPSC-CMs cultured without insulin (B27-) (Figure 3). The mitochondrial content per cell remained unchanged, suggesting that there was an increase in metabolic activity rather than mitochondrial development.

In our previous work we showed that supplementing hiPSC-CMs with a high dose of insulin (B27+, ∼58 nM) results in cell cycle activation via IGF–AKT–FOXO signaling. Here we show that culturing D12 hiPSC-CMs for 5 days with only a low dose of insulin (5–10 nM) does not increase the proportion of Ki67-positive cells, in contrast to hiPSC-CMs cultured in high dose insulin (B27+, 58 nM) (Figure 4).

Supplementation of low dose insulin (1 : 10 B27+/B27-) enhanced metabolism, while preventing upregulation of cell cycle engagement. Therefore, we recommend to use low dose insulin (1 : 10 B27+/B27-) over no insulin (B27-) during maturation of hiPSC-CMs.

## Limitations

The simple strategy for maturation as suggested in this protocol, despite increasing the mRNA expression of mature sarcomeric genes, is not sufficient to induce actual functional sarcomeric maturation on a protein level (ssTnI for cTnI, or MYH7 for MYH6 substitution). Advanced maturation strategies should be applied to achieve actual functional maturity such as engineered heart tissues (EHTs), co-culture, electric stimulation, and/or maturation medium. However, this approach offers a simple strategy to rapidly induce cell-cycle exit and set the stage for further downstream maturation.

## Troubleshooting

### Problem 1

Phenotype of hiPSC-CMs after *Step 1: Differentiation Toward hiPSC-CMs* is not comparable to the example video (Methods video S1).

### Potential solution

Possible solution: Each cell line needs optimization of the CHIR99021 concentration for optimal induction of mesoderm at D0 of the differentiation protocol (sub-step 2a). Use a gradient of CHIR99021 from 4 to 10 μM in a 12 well plate and fix cells at D2 using 4% PFA. Stain for Brachyury T and DAPI to determine the degree of mesoderm induction and continue differentiation with the most optimal concentration.

### Problem 2

After *Step 1: Differentiation Toward hiPSC-CMs*, differentiation is impure or displays <80% contractile area.

### Potential solution

Potential solution 1: First check whether the well is infected by looking at the color indicator (yellow indicates that the medium is acidic, indicative of an infection) and the transparency of the culture media. Cloudy media may also indicate an infection. Look at the bottom of the culture plate with high magnification to identify possible bacteria. It is also advised to test your cell culture once per month for mycoplasma.

Potential solution 2: Make sure confluency of hiPSCs is around 85–90% before initiating differentiation (Figure 2). Too high or too low confluencies may hamper differentiation efficiency.

Potential solution 3: Optimize the concentration of CHIR99021 that is used for mesoderm induction (4 – 10 μM) (sub-step 2a).

Potential solution 4: If no infection is present and potential solutions 1, 2 and 3 do not help, Step *2: Purification (<80% purity)* can be performed. A faster approach to increase the purity of your differentiation is to perform simultaneous expansion (*sub-step 3a*).

### Problem 3

There is only little or no proliferation during *Step 5: Expansion*.

### Potential solution

Possible solution 1: Make sure to use CHIR99021 that is freshly thawed. Also, consider optimizing CHIR99021 concentration between 2 to 5 μM for optimal proliferation efficiency for your specific cell line.

Possible solution 2: Replating density is too dense causing proliferation to halt. Make sure to use a replating ratio of ∼1 : 8 to make ensure absence of cell-cell contacts.

Possible solution 3: Cells may be too old or have reached expansion limit. Expansion works most optimally for hiPSC-CMs immediately following differentiation ∼D9. Culturing hiPSC-CMs for longer durations lowers their expansion capacity. Also, after three rounds of expansion the proliferation rate drops significantly.

### Problem 4

Cells are not viable during *Step 6: Maturation*.

### Potential solution

Possible solution 1: To prevent metabolic impairment after the abrupt loss of insulin from the culture media, supply hiPSC-CMs with low dose insulin (sub-step 12).

Possible solution 2: Consider using pen strep (P/S) to prevent the occurrence of an infection.

## Resource availability

### Lead contact

Further information and requests for resources and reagents should be directed to and will be fulfilled by the lead contact, Jan Willem Buikema (j.w.buikema@amsterdamumc.nl).

### Technical contact

Technical questions on executing this protocol should be directed to and will be answered by the technical contact, Devin Verbueken (d.l.verbueken@amsterdamumc.nl).

### Materials availability

This study did not generate new unique reagents.

### Data and code availability

Original data underlying this protocol are available at https://doi.org/10.1016/j.stemcr.2024.11.001. All associated RNA-seq data were deposited under GEO: GSE278598.

## Acknowledgments

We thank Joseph C. Wu from Stanford University for providing hiPSC lines SCVI-111, SCVI-114, and SCVI-273. This work was funded by the Dutch Heart Foundation Dekker Senior Clinical Scientist grant (J.W.B.) The graphical abstract was created with BioRender (J.W.B.; https://BioRender.com/kcs5eg0).

## Author contributions

Manuscript writing, D.V. and J.W.B.; experimental design, procedure, data analysis, and Figure 3, D.V. and V.A.J.W.; Figure 4, D.V.; differentiation of hiPSC-CMs, D.V., Q.Y., and S.A.; video material K.Z.; images L.J.M.K.; manuscript review and editing, Q.Y., V.A.J.W., L.J.M.K., J.v.d.V., L.B., S.A.J.C., and J.W.B. All authors have read and agreed to the publication of the final version of the manuscript.

## Declaration of interests

The authors declare no competing interests.

## Supplemental Information

Methods video S1. Highly pure and contractile hiPSC-CMs after differentiation, related to sub-step 2 from step 1: Differentiation Toward hiPSC-CMs

Methods video S2. Homogenous monolayer of hiPSC-CMs, related to sub-step 6 of step 3: Replating
